# Supplementary material for: In-situ diagnostic of femtosecond laser probe pulses for high resolution ultrafast imaging
Source: Light Sci Appl. 2021 Jun 16;10:126. doi: 10.1038/s41377-021-00562-1 (PMC8209123; doi:10.1038/s41377-021-00562-1)
Supplement: Supplementary file 1 — Supplemental Material [file 41377_2021_562_MOESM1_ESM.pdf]

Supplementary Information for

In-situ diagnostic of femtosecond laser probe pulses for high resolution ultrafast imaging

Chen Xie,[1,2*] Remi Meyer,[2*] Luc Froehly,[2] Remo Giust,[2] Francois Courvoisier,[2**]

[1]Ultrafast Laser Laboratory,
Key Laboratory of Opto-electronic Information Technology of Ministry of Education,
School of Precision Instruments and Opto-electronics Engineering,
Tianjin University, 300072 Tianjin, China

[2]FEMTO-ST institute, Univ. Bourgogne Franche-Comté, CNRS,
15B avenue des Montboucons, 25030, Besançon Cedex, France

[*] Equal contribution.
[**] Corresponding author francois.courvoisier@femto-st.fr

1 Parameters for the experiments shown in the supplementary figures

	Fig. S1	Fig. S3	Fig. S4	Fig. S5
Material	Air	FS	Sapphire	FS
E_{pump} (μJ)	$<$ 1 nJ	0.5	0.5	0.1
θ (°)	22	15	12	15

Table S1: Parameters used in the experiments shown in the figures above: material, pump energy E_{pump} and half-crossing angle of the pump beams θ. In all cases, to match the Bragg incidence angle, α is half of θ because the probe central wavelength is half of the pump one. FS stands for fused silica.

All angles are given in material and correspond to a single grating pitch $\Lambda = 1.1$ μm.

2 Characterization of pump and probe beams

Figure S1 shows the pump and probe beam characterizations in air. The pump beam is shaped by the SLM to generate an interference pattern as described in section 2, with $\theta = 22°$ and $\alpha = 11°$ in air. The grating period is therefore Λ = 1.1 μm. Experimental characterization and simulations of the spatial distribution are in excellent agreement. The probe beam is a homogeneous Gaussian beam. Our characterization shows that it is slightly wider than the fringe pattern and actually well superimposed over the fringes structure of the pump beam (in Fig. S1, x and z scales are absolute distances, identical for the pump and probe beams).

Technically, the experimental characterization of the near field is performed after replacing the last imaging lens of the setup described in Fig. 1(d) by another convex lens with twice the focal length so as to image the near field of the focal plane of the microscope objective on the camera.

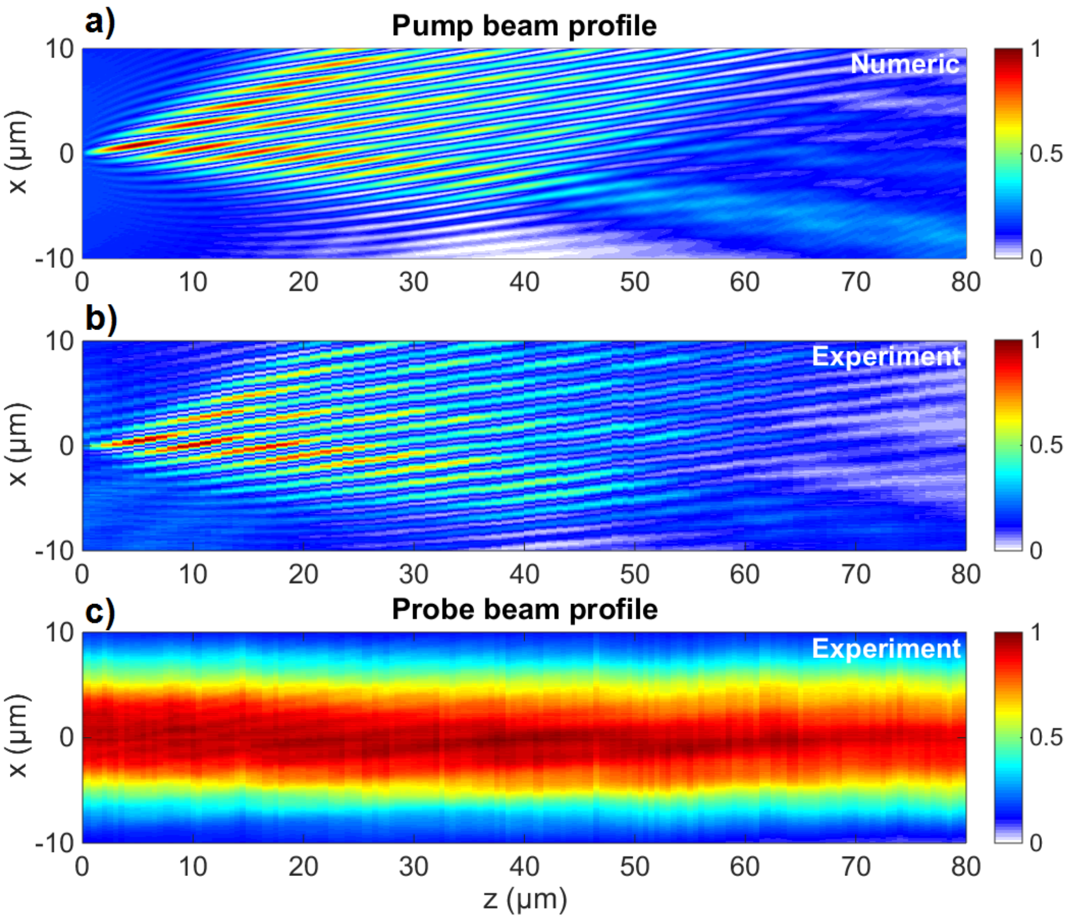

Figure S1: Fluence distribution in air of pump and probe beams along the propagation direction , *i.e.*, in (x, z) plane for $y = 0$. (a) Numerical simulation for the pump beam using beam propagation method (b) Experimental characterization of the pump beam. (c) Experimental characterization of the unperturbed probe beam, in absence of pump.

3 Comparison of cross-correlation signals for different pump energies

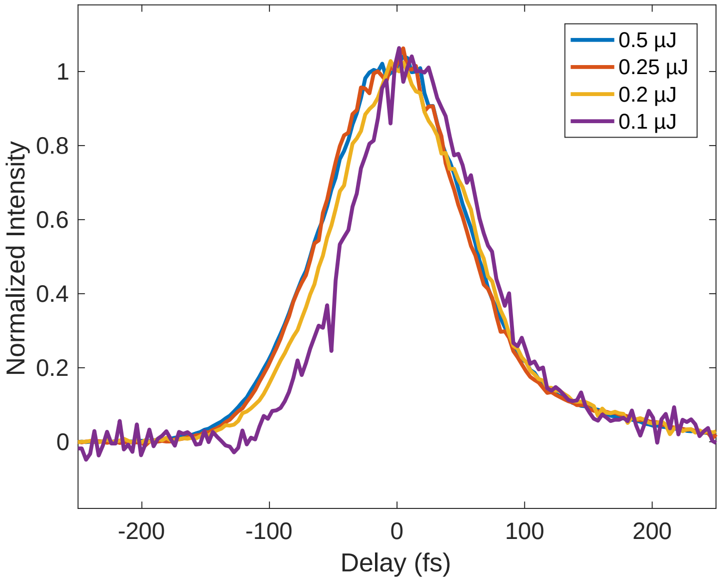

Figure S2: Comparison of cross-correlation traces obtained with increasing pump energy

Here we have plot the curves of the inset of Fig.2 normalized to 1. We see that for the lowest pulse energy, the signal slightly shifts towards larger delays. This can be explained by the non-uniformity of the intensity in the interference pattern (see Fig. S1). At very low intensities, only the onset of the interference is efficient in the diffraction, while at larger intensities, most of the diffracted signal originates from a region 20 µm away. Therefore, at the lower energy, the blue pulse travels less through the sample to meet the transient grating.

In all other acquisitions, we used the same 0.5 µJ pulse energy.

4 Cross-correlation curve for a non-optimal dichroic filter

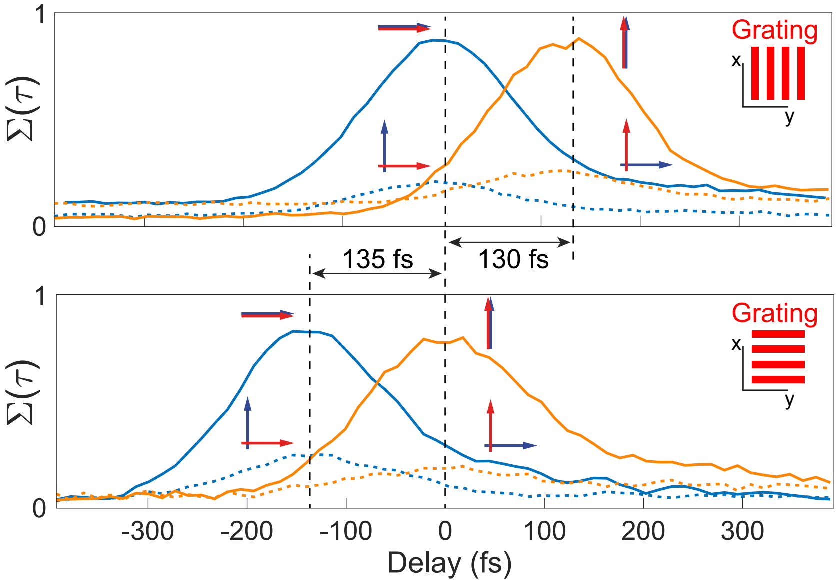

Figure S3: Evidence of a 133±3 fs shift induced by an imperfect dichroic mirror used for the pump-probe beams recombination. Blue and red arrows show respective polarization states of probe and pump beams.

This section shows a typical example where our technique shows to be particularly useful to identify potential flaws in the pump-probe timing. Here, the experiment is the same as described in the main text, except that the dichroic mirror shown in Fig. 1(d) is a model Thorlabs DMSP650 instead of Layertech 101495. Figure S3 shows the diffracted intensity as a function of the pump-probe delay for all polarization combinations. This is presented, on top, for a configuration of the laser-induced grating where the periodicity is along y-direction. This is repeated in the bottom figure for a periodicity along x direction, as in the results shown in the main text (see Fig. 2(b)).

The solid lines show the results for collinear pump and probe polarizations. Dashed lines are used for orthogonal polarizations. We observe that in all cases, the signal for a horizontal pump polarization (blue curves) leads to a delay of approximately 130 fs with respect to the vertical one. This experiment shows that the dichroic mirror induces a distortion of the spectral phase for the vertical pump polarization important enough to make a retardance exceeding the pulse duration. In contrast, Fig. 2(b) in the main text shows the results in the same conditions for a different dichroic mirror (Layertech 101495) where the shift is absent. We note that detecting this discrepancy between vertical and horizontal polarizations would be extremely difficult without our technique.

5 Compression of the probe pulse

The cross-correlation signal provided by the transient grating allows for controlling the probe compression and estimating the probe pulse duration.

In figure S4, we plot the diffracted signal as a function of delay for different positions of the prism compressor (insertion of glass in the probe beam path). We observe that for the position noted as “0 mm”, the cross-correlation signal is the most compressed. This corresponds to the shortest pulse duration achievable for the probe pulse. Using the knowledge of the pump pulse duration ($\simeq$ 110 fs), we retrieve the probe pulse duration by numerical fitting of the experimental curve, which leads to a probe pulse duration of $\simeq$ 60 fs.

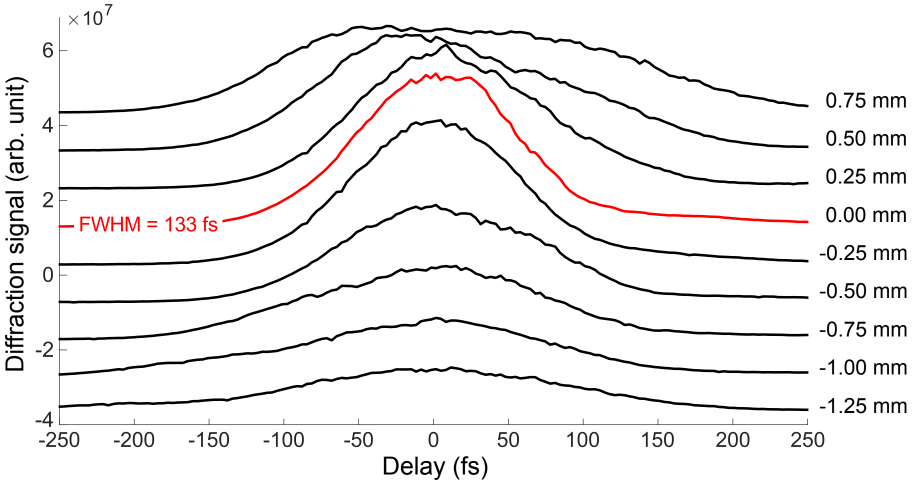

Figure S4: Signal for different positions of the prism insertion.

6 Synchronization in fused silica

Figure S5 shows, as in the main text Fig 3, the evolution of the diffracted signal as a function of the pump-probe delay for three different positions of the sample. This time, it is performed for fused silica material instead of sapphire. We obtain similarly an excellent agreement between experimental data and the prediction of Eq.2, *i.e.*, -22.6 fs for 100 μm.

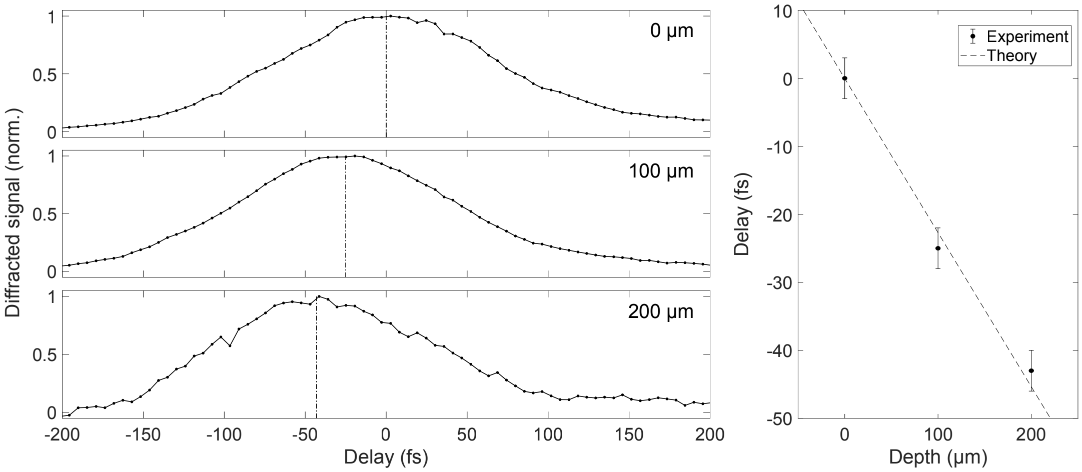

Figure S5: (left) Evolution of the diffracted signal as a function of sample position in fused silica (from 0 to 200 μm). (right) Barycenter of the diffracted signal as a function of sample displacement.

7 Inclination of the t-k_y traces of Fig. 5 in presence of angular dispersion

In this section, we analytically derive the inclination angle of the traces of Fig. 5. We start with the expression of a pulse with a Gaussian distribution in space and time, with waist w_0 and with pure angular dispersion with parameter $p = \frac{\mathrm{d}k_y}{\mathrm{d}\omega}$[57] in the direction y and second order phase ϕ_2. The pulse duration (FWHM) is $\sqrt{2log(2)}T$:

$$\tilde{E}(y,\omega) = E_0 e^{-T^2\omega^2/4} e^{-x^2/w_0^2} e^{-ip\omega y} e^{i\phi_2/2\omega^2} \tag{11}$$

In this equation, t corresponds to the pump probe delay.

After double Fourier transformation over y and t coordinates:

$$\hat{E}(k_y,t) = E_0' e^{-\alpha t^2 - \frac{(k_y - 2i\alpha p t)^2}{4(1/w_0^2 + p^2\alpha)}} = E_0' e^{u+iv} \tag{12}$$

with E_0' being a constant, u and v real-valued, and

$$\alpha = \frac{1}{T^2 + 2i\phi_2} = \alpha' + i\alpha''$$

We isolate u:

$$\begin{aligned} u = -\alpha' t^2 - &\frac{1}{\Delta}\Big[k_y^2 - 4(pt)^2(\alpha'^2 - \alpha''^2) + 4k_y p t \alpha''\Big] \times \Big[\frac{1}{w_0^2} + p^2\alpha'\Big] \\ &+ \frac{(p)^2\alpha''}{\Delta}\Big[-4k_y p t\alpha' - 8\alpha'\alpha'' p t\Big] \end{aligned} \tag{13}$$

with $\Delta = 4\Big[\Big(\frac{1}{w_0^2} + p^2\alpha'\Big)^2 + \Big(p^2\alpha''\Big)^2\Big]$.

The location of the iso-intensity patterns detected on the camera is determined by $u = K$, where K is a constant. This equation can be rewritten in a quadratic form, which is the equation of an ellipse. We introduce the normalized transverse wavevector $\tilde{k_y}$ and time $\tilde{t}$ with $k_y = \kappa\tilde{k_y}$ and $t = \tau\tilde{t}$.

$$A\tilde{k_y}^2 + B\tilde{k_y}\tilde{t} + C\tilde{t}^2 + D\tilde{k_y} + E\tilde{t} + F = 0 \tag{14}$$

with the following values:

$$
\begin{aligned}
A =& -\frac{\kappa^2\xi}{\Delta} \\
B =& \kappa\tau\frac{(4p\alpha''\xi - 4p^3\alpha'\alpha'')}{\Delta} \\
C =& \tau^2\frac{(\Delta\alpha' + 4p^2\alpha''^2\xi - 4p^2\alpha'^2\xi - 8\alpha'\alpha''^2p^4)}{\Delta} \\
D =& 0 \\
E =& 0 \\
F =& 0
\end{aligned}
\tag{15}
$$

F is set to zero but it can be any constant and $\xi = \left(\frac{1}{w_0^2} + p^2\alpha'\right)$.

Equation 14 describes an ellipse in (k_y, t) space, which can be rewritten in a matrix form as:

$$
{}^T X \mathbb{A} X +^T \mathbb{B} X + F = 0 \tag{16}
$$

with

$$
\begin{aligned}
X &= \begin{pmatrix} k_y \\ t \end{pmatrix} \\
\mathbb{A} &= \begin{pmatrix} A & B/2 \\ B/2 & C \end{pmatrix} \\
\mathbb{B} &= \begin{pmatrix} D \\ E \end{pmatrix}
\end{aligned}
$$

The major axis of the ellipse in (k_y, t) space is rotated by an angle θ from the k_y axis. What follows is the determination of this angle. The rotation matrix $R = \left(\begin{smallmatrix} \cos\theta & -\sin\theta \\ \sin\theta & \cos\theta \end{smallmatrix}\right)$ is chosen so that ${}^T R \mathbb{A} R$ is diagonal. The two eigenvalues of $\mathbb{A}$ are:

$$
\lambda_{1,2} = \frac{A + C \pm \sqrt{A^2 + B^2 + C^2 - 2AC}}{2} \tag{17}
$$

The eigenvectors allow the construction of the rotation matrix R. Then the rotation angle can be determined from:

$$
\tan\theta = \frac{-B/2}{C - \lambda_2} = \frac{B}{A - C + \sqrt{A^2 + B^2 + C^2 - 2AC}} \tag{18}
$$

In the following, we perform a development of A,B and C assuming that the temporal dispersion

ϕ_2 is large and the angular dispersion p is small. We use the following adimensional parameter:

$$\varepsilon = \frac{p^2 T^2 w_0^2}{\phi_2^2}$$

After a lengthy but straightforward calculation, we get at the first order in ε:

$$\begin{aligned} A \simeq & \frac{\kappa^2 w_0^2}{4}\Big(-1+\frac{\varepsilon}{4}\Big) \\ B \simeq & \kappa\tau\Big(1-\frac{\varepsilon}{4}\Big)\frac{p w_0^2}{2\phi_2} \\ C \simeq & \frac{\tau^2}{4T^2}\Big[-\frac{T^4}{\phi_2^2}+\varepsilon\Big(-1+\frac{5\tau^4}{4\phi_2^2}\Big)\Big] \end{aligned} \tag{19}$$

We then find the denominator of Eq. 18:

$$A - C + \sqrt{A^2+B^2+C^2-2AC} \simeq \frac{\tau^4 T^4}{8\kappa^2 w_0^2 \phi_2^4} + \varepsilon\Big(\frac{\tau^2}{2T^2} - \frac{\tau^2 T^2}{8\phi 2^2} + \frac{\tau^4}{4\kappa w_0^2 \phi_2^2}\Big) \tag{20}$$

This can be further simplified using the fact that $\phi_2 \gg T^2$ such that

$$A - C + \sqrt{A^2+B^2+C^2-2AC} \simeq \frac{\tau^2}{2T^2}\varepsilon \tag{21}$$

We finally get:

$$\tan\theta \simeq \frac{\kappa}{\tau}\frac{\phi_2}{p} \tag{22}$$
